# Cardiac Involvement in Glycogen Storage Disease Type IV: Two Cases and the Two Ends of a Spectrum

**DOI:** 10.1155/2012/764286

**Published:** 2012-09-26

**Authors:** Tolga Aksu, Ayse Colak, Omac Tufekcioglu

**Affiliations:** ^1^Department of Cardiology, Kocaeli Derince Education and Research Hospital, 41900 Kocaeli, Turkey; ^2^Department of Cardiology, Ankara Yuksek Ihtisas Education and Research Hospital, Sihhiye, 06410 Ankara, Turkey

## Abstract

Glycogen storage disease type IV (GSD IV) is an autosomal recessive disorder due to the deficiency of **α** 1,4-glucan branching enzyme, resulting in an accumulation of amylopectin-like polysaccharide in various systems. We describe two cases, a 23-year-old girl with dilated cardiomyopathy who presented with progressive dyspnea and fatigue and a 28-year-old girl with hypertrophic cardiomyopathy who was asymptomatic, secondary to the accumulation of amylopectin-like fibrillar glycogen, in heart. In both patients, the diagnosis was confirmed by enzyme assessment. Our patients showed that GSD IV is not only liver or skeletal muscle disease, but also it can be presented in different form of the spectrum of cardiomyopathy from dilated to hypertrophic and from asymptomatic to decompensated heart failure. Also, to our knowledge, this is the first hypertrophic cardiomyopathy case due to GSD IV in the literature.

## 1. Introduction

Glycogen storage disease type IV (GSD IV) is an autosomal recessive disorder due to a deficiency of glycogen branching enzyme (GBE) [1]. The branching enzyme catalyses the last step in glycogen biosynthesis by attaching short glucosyl branches in a-1,6-glycosidic linkages to linear chains of nascent glycogen [2] and the deficiency results in the accumulation of abnormal glycogen, with fewer branching points, and amylopectin-like polyglucosans in different tissues to various degrees [1].

Many variants of GSD IV with different tissue involvement and variable clinical manifestations have been reported. Typically, the presentation is in childhood with liver involvement often leading to cirrhosis, with death by 5 years of age; some patients, however, have mild liver disease without progression and can reach adulthood without liver transplantation [3]. In multiple system involvement, the deficiency of the enzyme was detected in both muscle and the liver [4]. This includes peripheral myopathy with or without cardiomyopathy and neuropathy [5]. The age of onset ranges from neonatal to adult age.

We report two female cases with GSD IV, one of whom has dilated cardiomyopathy, and the other one has hypertrophic cardiomyopathy.

## 2. Cases

**Case 1**

**Figure 1:**
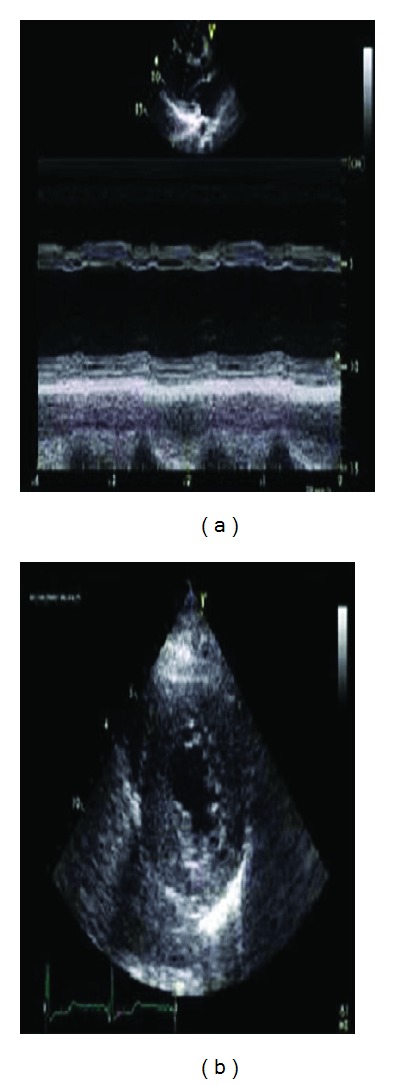
(a) Parasternal long-axis view showing the left ventricular dilatation and advanced global hypokinesis. (b) Parasternal short axis view showing the septal and posterior wall hypertrophy.

A 23-year-old woman was admitted to hospital for further examination of cardiomegaly following an abnormal telecardiography. She had been well until 14 months ago when she suffered progressive dyspnea (New York Heart Association, class 3) and fatigue. None of her relatives had a history of heart failure or glycogenosis. On her medical history, she admitted to hospital with jaundice and laboratory tests showed hemoglobin 11 g/dL, white blood cell count 9,000/mm^3^ (neutrophils 70%, lymphocytes 22%, monocytes 7%, and eosinophils 1%), platelet count 155,000/mm^3^, total protein 6.7 g/dL, albumin 3.1 g/dL, aspartate aminotransferase 552 IU/L, alanine aminotransferase 241 IU/L, total bilirubin 4.9 mg/dL, direct bilirubin 2.8 mg/dL, gamma-glutamyl transpeptidase 94 IU/L, and alkaline phosphatase 320 IU/L. She underwent living donor liver transplantation from her father, a heterozygous donor. The transplant was uneventful. Microscopic examination revealed variable-sized regenerating nodules, supporting the diagnosis of cirrhosis. The intracytoplasmic inclusions were strongly positive on periodic acid-Schiff (PAS) staining, but were resistant to diastase, excluding other types of glycogen storage disease. Electron microscopy demonstrated that the cytoplasmic inclusions contained undulating, randomly oriented, and delicate fibrils, confirming the diagnosis of glycogen storage disease type IV. Following liver transplantation, the patient was treated with standard immunosuppressive therapy. Pretransplantation echocardiography was entirely normal. Upon present admission, a grade 2 pansystolic murmur was heard at the apex. She manifested bibasilar rales and bilateral legs edema. Routine laboratory studies were normal. The first documented electrocardiography (ECG), before transplantation had shown no abnormality but ECG in admission revealed ST depression in leads V5-6 and the left atrial dilatation in leads V1-2. The cardiothoracic ratio was 61%. Echocardiography showed globally advanced hypokinetic motion in the left ventricle (LV). LV end-diastolic dimension was 54 mm and LV ejection fraction was 18% (Figure 1(a)). In cardiac catheterization, the mean pulmonary capillary wedge pressure was 21 mmHg, left ventricular end-diastolic pressure was 20 mmHg, and the cardiac index was 1.9 L/min^−1^/m^−2^ on cardiac catheterization.

**Figure 2:**
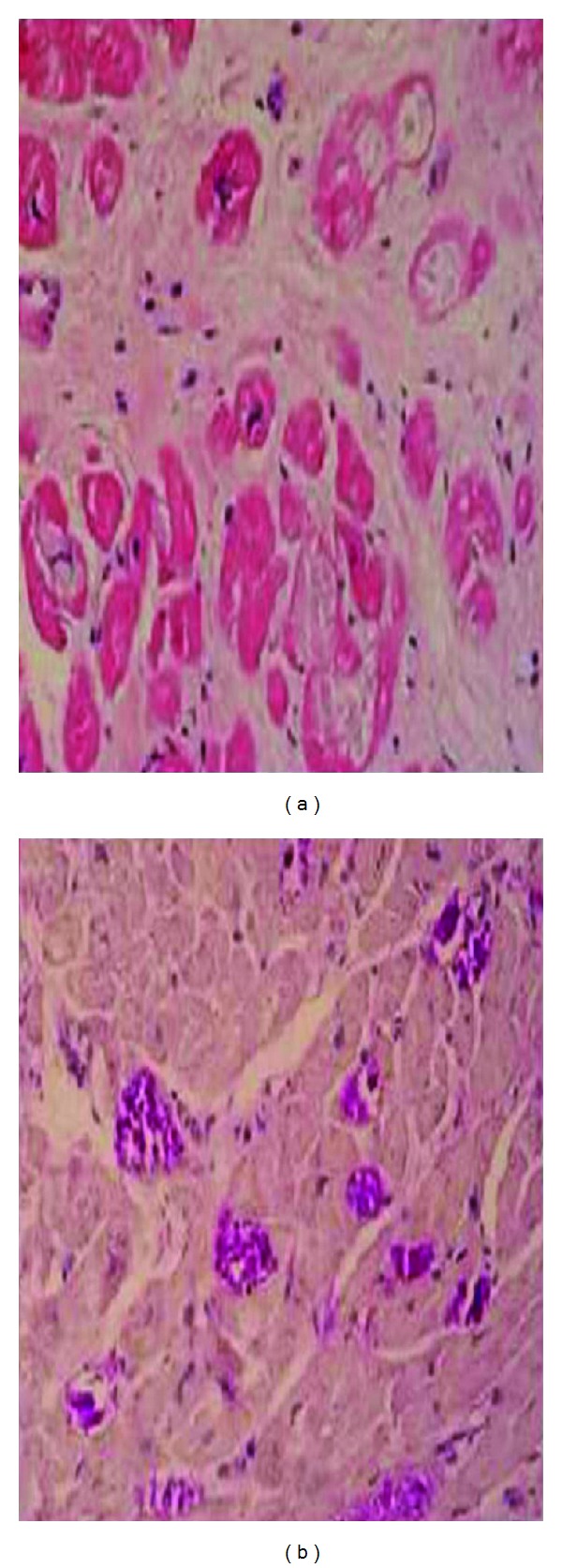
(a) Microscopic findings of the heart showing pale cytoplasmic inclusion-like material within cardiac myocytes; hematoxylin/eosin, ×200. (b) Periodic acid-Schiff (PAS)-positive, large cytoplasmic inclusions in cardiac myocytes, ×200.

LV endomyocardial biopsy specimen demonstrated central vacuolar degeneration of myocytes with depositions of PAS-positive granules (Figure 2), which proved to be glycogen, because they were digested with diastase. Myocardial histology revealed increased interstitial fibrosis and replacement fibrosis.

The patient was treated with enalapril 2 × 5 mg/day, metoprolol 50 mg/day, spironolactone 25 mg/day, and furosemid 20 mg/day. The patient was discharged in the end, two weeks after her admission early and followed up as an outpatient. She has not experienced any symptoms of heart failure at the 6 months of her followup.

**Case 2**

A 28-year-old woman presented to our clinic with asymptomatic left ventricular hypertrophy in ECG. The patients had undergone liver biopsy twice and diagnosed as GSD IV when she was in childhood. The patients had hypoglycemia attacks once a month in history. Laboratory findings showed hypoglycemia (2.1 mmol/liter) at fasting, without any symptoms of hypoglycemia.

She had no history of liver transplantation and she was on diet therapy only. She had no family history of a metabolic abnormality. On presentation, the patient had stable vital signs. The physical examination revealed that the patient had minimal hepatomegaly but not splenomegaly and the other systems were thoroughly normal. ECG revealed concentric left ventricular hypertrophy, and the echocardiogram findings were consistent with hypertrophic cardiomyopathy (Figure 1(b)).

As the patient was asymptomatic and there was no risk factor for sudden death, the patient discharged without any medical treatment and followed up as an outpatient. She has not experienced any symptoms of heart failure at the 6 months, 1 year, and 2 years of her follow up.

## 3. Discussion

GSD IV arises in case of a deficiency of GBE activity and is also referred to as amylopectinosis since the accumulated glycogen is abnormal and has longer chain lengths and fewer branch points, resulting in a structure resembling amylopectin [1]. The abnormal glycogen accumulates in all tissues, particularly in the liver, skeletal muscle, and myocardium [2, 3]. Although progressive liver cirrhosis is the classic and most common clinical presentation for GSD IV, a review of the literature demonstrates significant clinical variability [6].

In our first case, the diagnosis of dilated cardiomyopathy due to GSD IV was confirmed by heart biopsy. The biopsy showed intracellular, basophilic, diastase resistance, PAS-positive inclusion bodies (polyglucosan bodies), and GBE deficiency. Although heart transplantation was planned in the case, it could not be performed as yet because of available donor was not found.

Patients with type IV GSD require a complete heart, liver, and muscle evaluation before considering transplantation. As there is no way to replace the deficient enzyme activity, liver transplantation is the only known treatment modality. It needs to be remembered that transplantation may only improve the hepatic aspect of GSD, while in patients with GSD IV, it may progress in other organs, namely the heart, skeletal muscle, and nervous system, which may be deleterious [7] as our case. To date, only 17 patients have been reported to receive liver transplants for this condition, and most did not develop cardiopathy or myopathy [8]. Three of these patients received transplants from living, related, heterozygous donors, as did our patient, and no mortality or morbidity related to heterozygosity has yet been observed. Extrahepatic deposits of polyglucosan have been reported to be resorbed through the migration of donor cells (forming a microchimerism) from the liver allograft [9]. Therefore, although liver transplantation was successful in our patient, long-term followup is necessary to guard against neuromuscular complications. 

Case 2 is a GSD IV patients complicated with hypertrophic cardiomyopathy and liver involvement without liver cirrhosis. The histopathology of the liver biopsy taken in childhood, which included positive glycogen staining, negative diastase PAS staining, glycogenated nuclei, increased glycogen content, and branching enzyme deficiency and she had been diagnosed as GSD IV.

GSD IV is a rare cause of amylopectinosis-associated cardiomyopathy with or without involvement of other organs. In particular, multiple system involvement including heart has been reported in only three cases in adult age [3, 10, 11]. These cases included dilated cardiomyopathy, liver involvement with or without involvement of other organs. Although the deficiency of GBE had been detected in both muscle and liver biopsy, no enzyme studies had been reported in any of the cases. We confirmed our diagnosis in with enzymatic study in cardiac biopsy specimen different from previous literature.

Although Danon's disease, an X-linked recessive lysosomal glycogen storage disease, progresses with hypertrophic cardiomyopathy and neurological involvement [12], there was no case of hypertrophic cardiomyopathy related to GSD IV in the literature in our knowledge. Nevertheless, the diagnosis of GSD IV was only confirmed with liver biopsy, heart biopsy was not performed. So whether cardiac involvement associated with GSD IV can not be proved with this findings in our case.

Our cases highlight that in a young patient who presents with dilated or hypertrophic cardiomyopathy, one should keep in mind uncommon causes such as GSD. Long-term prognosis of cardiac involvement in GSD IV has not known yet. The studies on genetic and clinical features of new cases, especially with heart involvement, should allow us to understand the disease of multisystemic involvement secondary to GSD and prognosis of the disease.
